# Galectin-1 and -3 in high amounts inhibit angiogenic properties of human retinal microvascular endothelial cells *in vitro*

**DOI:** 10.1371/journal.pone.0265805

**Published:** 2022-03-23

**Authors:** Anna Hillenmayer, Christian M. Wertheimer, Arie Geerlof, Kirsten H. Eibl, Siegfried Priglinger, Claudia Priglinger, Andreas Ohlmann

**Affiliations:** 1 Department of Ophthalmology, University Hospital, LMU Munich, Munich, Germany; 2 Department of Ophthalmology, University Hospital Ulm, Ulm, Germany; 3 Protein Expression and Purification Facility, Institute of Structural Biology, Helmholtz Center Munich for Environmental Health, Neuherberg, Germany; Medical College of Georgia at Augusta University, UNITED STATES

## Abstract

**Purpose:**

Galectin-1 and -3 are β-galactoside binding lectins with varying effects on angiogenesis and apoptosis. Since in retinal pigment epithelial cells high amounts of human recombinant galectin (hr-GAL)1 and 3 inhibit cell adhesion, migration and proliferation, we investigated if hr-GAL1 and 3 have homologous effects on human retinal microvascular endothelial cells (HRMEC) *in vitro*.

**Methods:**

To investigate the effect of galectin-1 and -3 on HRMEC, proliferation, apoptosis and viability were analyzed after incubation with 30, 60 and 120 μg/ml hr-GAL1 or 3 by BrdU-ELISA, histone-DNA complex ELISA, live/dead staining and the WST-1 assay, respectively. Further on, a cell adhesion as well as tube formation assay were performed on galectin-treated HRMEC. Migration was investigated by the scratch migration assay and time-lapse microscopy. In addition, immunohistochemical staining on HRMEC for β-catenin, galectin-1 and -3 were performed and β-catenin expression was investigated by western blot analysis.

**Results:**

Incubation with hr-GAL1 or 3 lead to a decrease in proliferation, migration, adhesion and tube formation of HRMEC compared to the untreated controls. No toxic effects of hr-GAL1 and 3 on HRMEC were detected. Intriguingly, after treatment of HRMEC with hr-GAL1 or 3, an activation of the proangiogenic Wnt/β-catenin signaling pathway was observed. However, incubation of HRMEC with hr-GAL1 or 3 drew intracellular galectin-1 and -3 out of the cells, respectively.

**Conclusion:**

Exogenously added hr-GAL1 or 3 inhibit angiogenic properties of HRMEC *in vitro*, an effect that might be mediated via a loss of intracellular endogenous galectins.

## Introduction

Most common causes of blindness in the developed world include neovascular diseases of the retina such as diabetic retinopathy in adults [1–3] and retinopathy of prematurity in children [1]. In both pathologies, a loss of retinal vasculature or an impaired development of intraretinal vasculature leads to retinal ischemia, which induces the expression of pro-angiogenic factors such as vascular endothelial growth factor (VEGF) [4]. In turn, the angiogenic factors promote pre-retinal neovascularization, which subsequently can cause severe complications such as vitreous bleedings, retinal detachment and fibrosis [5, 6]. Even though laser coagulation of the retina and intravitreal anti-VEGF therapy open up new possibilities to treat these pathologies, several limitations and adverse effects including neuronal degeneration are discussed [7, 8]. Therefore, novel therapeutic strategies are needed to overcome these limitations and adverse effects [9].

Galectins are lectins that can bind specifically to β-galactosylated protein, representing a common glycosylation motive of many different extra- and intracellular proteins as well as membrane proteins [10]. Even though galectin-1 as well as -3 are secreted proteins, and therefore localized in the extracellular matrix and at the outer surface of the cell membrane, both galectins can also be found in the cyto- and nucleoplasm [11–14]. Extracellular galectins can influence several signaling pathways via clustering of transmembrane receptors or by modulating cell-cell or cell-matrix interactions [15–17]. In the cytoplasm as well as in the nucleus, galectins can interact with specific proteins and hereby influence their functions or the transcription of specific target genes [18–20]. Via these various opportunities to modify cellular signaling, galectins can modulate tumor biology, immune and inflammatory responses, neural degeneration and wound repair [17, 21–23].

In the eye, enhanced galectin-1 expression is associated with diabetic retinopathy, where the galectins co-localize with the VEGF receptor in retinae and fibrovascular membranes [24]. In addition, increased galectin-1 level were detected in the aqueous humor of patients suffering from proliferative diabetic retinopathy and neovascular glaucoma [25]. Conforming with these clinical observations, endogenously expressed galectin-1 induces proliferation, migration, and vascular permeability in microvascular endothelial cells [26]. Furthermore, after induction of an oxygen-induced retinopathy, a model for retinopathy of prematurity in mice, galectin-1 was highly expressed in the retina and predominantly localized in neovascular tufts. Vice versa, in galectin-1 deficient mice, a reduced retinal neovascularization was observed following an OIR [25, 27]. Homologous observations were made in the absence of galectin-3 [28]. In addition, the inhibiton of endogenous galectin-3 by a small molecule led to a reduced corneal neovascularization and fibrosis in mice [29].

However, there is growing evidence that exogenously added galectin in high concentrations can have opposed effects to that of endogenous galectins. In a previous study, we observed that exogenous galectins in high amounts can block cell adhesion of retinal pigment epithelial cells [30]. Several other studies have correspondingly shown an inhibitory effect of exogenous galectin in diseases in which endogenous galectin was supposed to enhance pathological progression [17, 31–34]. Further on, an inhibitory effect of recombinant galectin-1 on the proliferation of human fibroblast, epithelial carcinoma cells, osteosarcoma cells and hematopoietic stem cells had been observed at concentrations between 10 and 160 μg/ml in previous studies [35, 36]. In humans, galectin-1 concentrations in blood plasma range between 20 ng/ml in healthy individuals and 650 ng/ml in patients suffering from epithelial ovarian cancer [37, 38]. Homologous concentrations are reported for galectin-3 ranging between 7 ng/ml for normal humans and 950 ng/ml in patients suffering from metastatic cancer [37, 39]. Thus, the inhibitory effects of exogenous galectins can be observed at much high concentrations than those of physiological as well as pathological conditions.

To evaluate whether exogenous galecins in high amounts have the potential to block angiogenesis *in vitro*, the influence of exogenous human recombinant GAL1 or GAL3 (hr-GAL1 or 3) on angiogenic properties of human retinal microvascular endothelial cells (HRMEC) were investigated.

## Methods

### Cell culture

Primary HRMEC (Pelobiotech GmbH, Planegg, Germany) were used in passage 2–6. Cultivation was performed under standard cell culture conditions in an incubator (Thermo Fisher Scientific, Waltham, MA) at 37°C and 5% CO_2_. HRMEC were cultured on uncoated cell culture dishes (Thermo Fisher Scientific) in microvascular endothelial cell growth medium (Pelobiotech) containing endothelial cell growth supplement (Pelobiotech), 2% fetal calf serum (FCS, Pelobiotech), 50 μl/ml penicillin and 50 μg/ml streptomycin. If not stated otherwise, HRMEC were exposed to 30 μg/ml, 60 μg/ml and 120 μg/ml of either hr-GAL1 or 3 in growth factor supplemented culture medium. As a positive control (pos co), cell culture medium with supplements and 2% FCS was used, whereas for the negative control (neg. co), cells were incubated in cell culture medium without supplements and without FCS. To block exocytosis or proteasomal protein degradation HRMEC were incubated for 12 h with either 10 μM brefeldin A, which inhibits exocytic trafficking, or 5 μM epoxomicin, which covalently binds catalytic subunits of the proteasome (both from Selleckchem, Houston, TX) as previously described for vascular endothelial cells [40, 41].

### Hr-GAL1 and 3

Hr-GAL1 and 3 where isolated after transfection of E-coli as previously described [15]. In brief, pETM-11/hgalectin1 and pETM-11/hgalectin3 (constructed by G. Stier, European Molecular Biology Laboratory) were transformed into the E. coli strain BL21(DE3) (New England Biolabs, MA) and cultured at 20°C in 2-liter flasks containing 500 ml ZYM 5052 auto-induction medium and 100 μg/ml kanamycin (Merck, Darmstadt, Germany) [42]. Cells were harvested by centrifugation after reaching saturation and resuspended in 30 ml lysis buffer (20 mM Tris-HCl, 150 mM NaCl, 10 mM MgSO_4_, 10 μg/ml DNase1, 1 mM AEBSF.HCl, 0.03% (v/v) CHAPS, 1 mg/ml lysozyme, pH 7.5), and lysed by sonication. Following centrifugation (40000 x g) and filtration (0.2 μm) of the lysates, supernatants were applied to 2-ml lactose-agarose columns (J-Oil Mills, Tokyo, Japan), which were equilibrated in buffer A (20 mM Tris-HCl, 150 mM NaCl, 0.03% (v/v) CHAPS, pH 7.5). After 3 washes with 25 ml buffer A, bound proteins were eluted two times with 5 ml buffer A containing 0.2 M β-lactose and dialyzed overnight at 4°C against 1 l 1xPBS. The dialysates were filtered (0.2 μm) and stored at 4°C. Protein concentration was determined by measuring the absorbance at 280 nm.

For biotinylation of hr-GAL1 and 3 the EZ-Link Sulfo-NHS-Biotin kit was used in accordance with the manufacturer’s recommendations (Thermo Fisher Scientific). In brief, 500 μg of hr-GAL1 or 3 were incubated at room temperature for 2 h with 80 μl or 40 μl of Sulfo-NHS-Biotin [10 mM], respectively. After labeling the protein solution was dialyzed against 1x PBS at 4°C over night to remove excessive biotin molecules using a Slide-A-Lyzer dialysis cassette with a cutoff of 3.5 kDa (Thermo Fisher Scientific).

### Immunohistochemistry

For immunohistochemistry, HRMEC were grown on round 15mm microscopy cover glasses (Thermo Fisher Scientific). After incubation, cells were washed 3 x 5 min with 1x phosphate-buffered saline (PBS; Biochrom, Berlin, Germany) and fixed with 4% paraformaldehyde (PFA) for 10 min. After 3 additional washings with 0.1 M phosphate buffer (1:1 Na_2_HPO_4_ x 2H_2_O + NaH_2_PO_4_ x H_2_O) for 5 min each, cells were incubated with blocking-solution (3% bovine serum albumin, 0.1% triton X-100 in 0.1 M phosphate buffer) for 1 h. After incubating overnight with rabbit anti-galectin-1 (Abcam, Cambridge, UK), rat anti-galectin-3 (Biolegend, San Diego, CA), rabbit anti-MCT-1 (Abcam) or rabbit anti-β-catenin (Cell Signaling, Danvers, MA) antibodies at a dilution of 1:50 in 1:10 diluted blocking-solution at 4°C, specimens were washed 3 times with 0.1 M phosphate buffer for 5 min each and incubated with 1:500 goat anti-rabbit Alexa Fluor 555, goat anti-rabbit Alexa Fluor 488 or donkey anti-rat Alexa Fluor 488 (all from Thermo Fisher Scientific) in 1:10 diluted blocking-solution for 2 h at room temperature. For visualization of biotinylated hr-GAL1 and hr-GAL3, specimens were incubated with 1:1000 streptavidin Alexa Fluor 488 in 1:10 diluted blocking-solution for 2 h at room temperature. Following additional three washings with 0.1 M phosphate buffer for 5 min each, cover slides were stained with Hoechst 33342 (Invitrogen) in a dilution of 1:2000 in 0.1 M phosphate buffer, washed again three times with 0.1 M phosphate buffer for 5 min each and mounted with antifade mounting medium (Vector Laboratories, Burlingame, CA). Immunofluorescence staining was analyzed on an Axio Observer 7 with an Apotome module to generate optical sections (Zeiss, Jena, Germany) and documented by using the ZEN software (Zeiss). For quantification of the intracellular fluorescent signal for galectin-1 or -3 of control and hr-GAL1 or 3 treated HRMEC images were taken using the Apotome module with the same setting conditions for one experiment. Densitometry was performed in the perinuclear cytoplasm using the circle tool of the ZEN software, which calculates the intensity of the fluorescent signal per area. The cytoplasm of 10 HRMEC per group and experiment was quantified and normalized to control cells.

### Cell proliferation and cell viability

Proliferation of HRMEC was determined using a 5-bromo-2’-deoxyuridine (BrdU) ELISA in accordance to the manufactures recommendations (Roche, Mannheim, Germany). In brief, 1.2x10^4^ cells/cm^2^ HRMEC were seeded onto a 96 well plate and incubated for 24 h to achieve complete adherence of the cells. After incubation with hr-GAL1 or 3 and BrdU labeling solution for 48 h, cells were fixed and incubated with anti-BrdU antibodies in accordance to the manufacturer’s instructions. Following the substrate reaction, the product formation was quantified by absorbance measurement at a wavelength of 450 nm and a reference at 690 nm on the SpectraMax 190 ELISA reader (Molecular Devices, San Jose, CA).

To analyze cell viability, a colorimetric dye reduction assay was conducted matching the manufacturer’s recommendations (WST-1; Roche). In brief, 1,5x10^4^ cells/cm^2^ were seeded and incubated for 24 h. After treatment of HRMEC with hr-GAL1 or 3 for 72 h, WST-1 was added and incubated for additional 2 h. For readout, absorbance measurement at a wavelength of 450 nm and a reference at 690 nm was determined on a SpectraMax 190 ELISA reader (Molecular Devices).

### Scratch migration assay and time lapse microscopy

A scratch migration assay was conducted as described before [43]. In brief, confluent HRMEC were scratched using a 100 μl pipette tip followed by debris removal by washing cells 2x with 1xPBS (Biochrom). Prior to incubation with hr-GAL1 and 3 as well as 24 h thereafter, the size of the wound area was documented using an inverted phase-contrast microscope (Leica DM IL, Leica, Wetzlar, Germany). For quantification, the wound area before and after treatment was measured using the Image J Version 1.52k (NIH, Bethesda, MD) and calculated as relative wound closure.

Time lapse microscopy was performed as described previously [44, 45]. Briefly, 1x10^3^ cells/cm^2^ HRMEC were seeded onto a 12 well plate and incubated for 24 h. Image documentation of the cell positions started within 10 min of treatment with an automated Axiovert100 inverted phase contrast microscope (Zeiss) with an incubator at 37°C and 5% CO_2_. Image documentation was performed every 10 min for a time period of 24 h. For single-cell tracking, the xy-positions of the cell at the specific time periods were tracked and recorded by Image J Version 1.52k (NIH) using a manual cell tracking software plugin (MTrackJ, kindly provided by Erik Meijering, University of Rotterdam, Netherlands). The data was then used for the reconstruction of trajectories from these coordinates, allowing the analyzation of single cell migration distance and mean velocity. The comparison of the coordinated behavior was conducted via a Rose plot, shifting the cell trajectories beginning points to the origin of the space [46].

### Cell adhesion assay

For the cell adhesion assay, HRMEC were seeded at 1x10^3^ cells/cm^2^ and incubated with hr-GAL1 or 3. Images were taken at the time periods of 15, 30, 60 and 120 min after seeding with a phase-contrast microscope (Leica). The number of attached cells was quantified and plotted as relative number of total cells.

### Tube formation assay

The tube formation assay was performed as previously described [43]. In brief, HRMEC were seeded on a matrix of extracellular basement membrane proteins (Cultrex, Trevigen, Gaithersburg, MD) in order to allow formation of capillary-like structures. After thawing, 156 μl Cultrex/cm^2^ were incubated for 30 min at 37°C with 4.7x10^4^ HRMEC cells /cm^2^ followed by incubation of hr-Gal1 and 3 for 4 h. After photo documentation, the formed tube area with a width of more than 30 μm was quantified by Image J 1.52k (NIH) and GNU Image Manipulation Program (GIMP) version 2.10 (The Gimp team, University of Berkley, California).

### Cell toxicity

To rule out any toxic effect of hr-GAL1and 3 on HRMEC, apoptosis and necrosis was evaluated. For the detection of apoptosis, an ELISA against histone-DNA complexes was performed after 72 h of incubation with 120 μg/ml of either hr-GAL1 or 3 (Cell Death Detection ELISA Plus, Roche) in accordance with the manufacturer’s recommendations. Absorbance was measured at a wavelength of 405 nm including a reference at 490 nm on the SpectraMax 190 ELISA reader (Molecular Devices).

To analyze late apoptosis and necrosis by live/dead staining, HRMEC were seeded on a cover slide and incubated with 120 μg/ml of either hr-GAL1 or 3 for 72 h or with methanol as positive control for 10 min. Following staining with 1 μg/ml Hoechst 33342 and 2 μg/ml propidium iodide (both from Thermo Fisher Scientific) in supplemented medium for 15 min, HRMEC were washed 3 times with 1xPBS (Biochrom) for 5 min each and fixed with 4% PFA for 10 min. After additional 3 washes with PBS for 5 min each, cover slides were mounted with antifade mounting medium (Vector Laboratories).

### Protein preparation and western blot analysis of β-catenin

For β-catenin Western blot analysis, confluent HRMEC were incubated with 120 μg/ml of either hr-GAL1 or 3 for 24 h. Cells were harvested and lysed in RIPA buffer containing protease and phosphatase inhibitor (Roche). After centrifugation to remove insoluble constituents, up to 15 μg total protein was subjected onto a 10% SDS-PAGE and transferred onto a PVDF membrane (Roche) by semidry blotting. After blocking with 5% low fat milk in PBS-T, the membranes were incubated overnight with rabbit-anti-β-catenin antibodies (Cell Signaling), diluted 1:1000 or goat-anti-α-tubulin antibodies (R&D System) diluted 1:5000 as loading control, in PBS-T with 0.5% BSA. AP-conjugated chicken-anti-rabbit or chicken-anti-goat antibodies were used as secondary antibodies at a 1:2000 dilution in PBS-T with 0.5% BSA. Antibody labeling was visualized using the CDP-Star substrate (Roche) and documented on an iBrightCL1000 Imaging System (Thermo Fischer).

### Statistical analysis

All graphs and calculations were done using EXCEL 365 version 16 (Microsoft, Redmond, WA), SPSS version 22 (IBM, Armonk, NY) and GraphPad PRISM 8 (GraphPad Software, Inc., San Diego, CA). Outliers, if present, were included in all calculations and analysis. For data presentation, the mean and the standard deviation was calculated. For comparison of the mean variables, a 1-way ANOVA was used. For data meeting the assumption of homogeneity of variances, a least significant difference (LSD) post hoc test was performed and for data not meeting the criteria, a Games Howell post hoc test. *P* values less than 0.05 were considered to be statistically significant. For the graphs, p values <0.05 were marked with *, p<0.01 with ** and p<0.001 with ***. All error bars are presented as the standard deviation.

## Results

### Effects of hr-GAL1 and 3 on intracellular localization of galectin-1 and -3 in HRMEC

First, we analyzed the effects of hr-GAL1 and 3 on localization of galectin-1 and -3 in HRMEC by immunohistochemistry.

**Fig 1:**
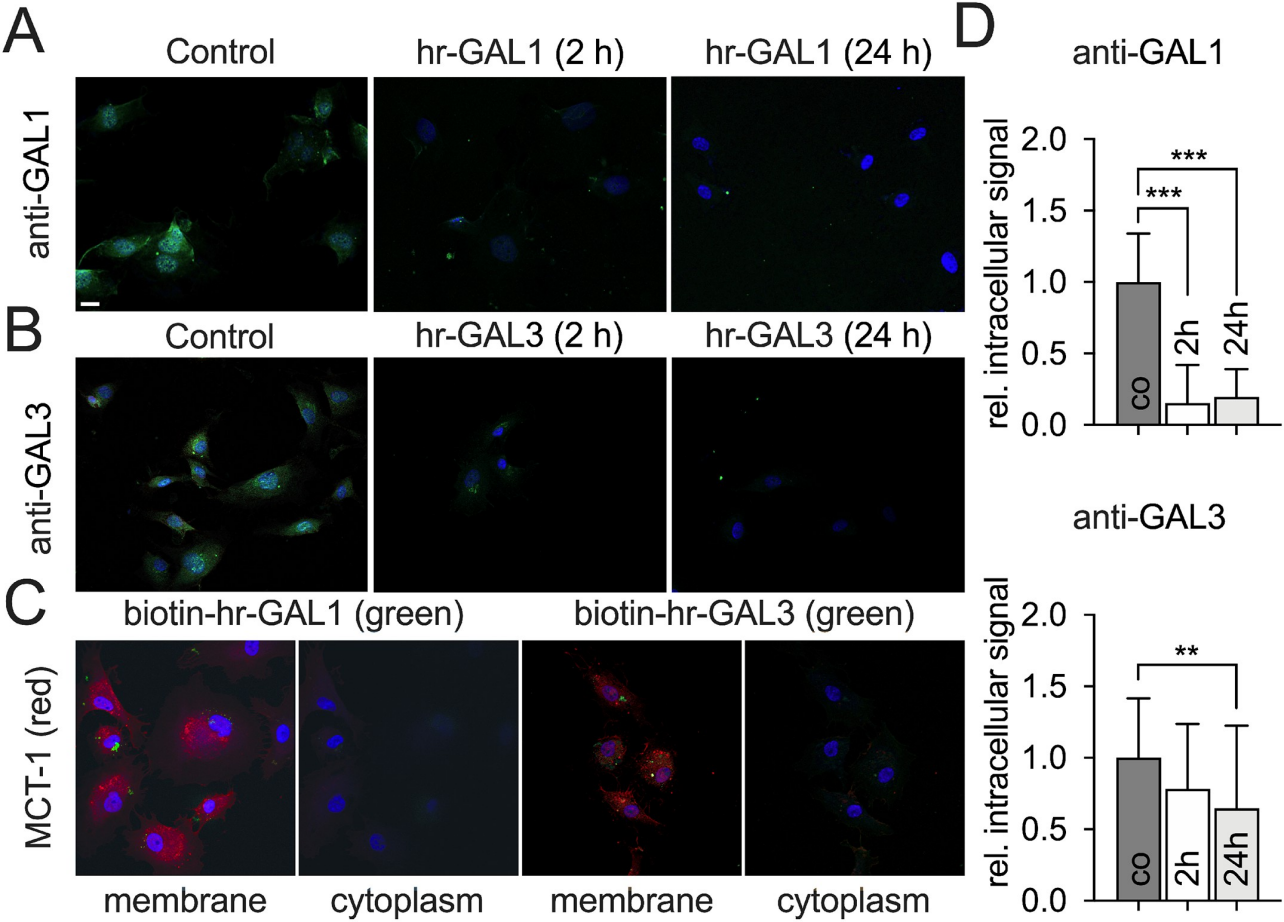
Localization of galectin-1 and -3 following incubation of HRMEC with hr-GAL1 and 3. Immunostaining of HRMEC for galectin-1 (A) and -3 (B) after incubation with 120 μg/ml hr-GAL1 and 3 for 2 as well as 24 h. C. Detection of biotinylated hr-GAL1 and 3 (green) after incubation of HRMEC with 30 μg/ml recombinant protein for 2 h and immunostaining for MCT-1 (red) as a marker for the cell membrane (C). Membrane, focal plane of the cell membrane; cytoplasm, focal plane of the cytoplasm; magnification bar in A–C, 20 μm; blue, Hoechst staining. D. The intracellular fluorescent signal for galectin-1 or -3 of the control group and hr-GAL1 or 3 treated HRMEC was quantified, normalized against the control group and plotted as relative intracellular fluorescent signal. **p<0.01; ***p<0.001; n = 30 of 3 independent experiments.

In control cells without incubation of hr-GAL1 or 3, endogenous galectin-1 and -3 was observed in the perinuclear cytoplasm and to a lesser extend in the nucleus. Intriguingly, following treatment of HRMEC with exogenous of hr-GAL1 or 3 for 2 h, the signal for galectin-1 and galectin-3 was markedly reduced in the cytoplasm as well as in the nucleus (Fig 1A and 1B). By quantification, a reduction of the cytoplasmic perinuclear fluorescent signal down to 16% for galectin-1 (0.16 ± 0.27, p<0.001, n = 30) and to 78% for galectin-3 (0.78 ± 0.45, p = 0.05, n>30) was observed. Following prolonged incubation of HRMEC with hr-GAL1 or 3 for 24 h, the intracellular level for galectin-1 remained very low (0.19 ± 0.19, p<0.001, n = 30) whereas for galectin-3, a further significant decrease to 64% was detected (0.64 ± 0.58, p = 0.002, n>30; Fig 1A, 1B and 1D).

To analyze if hr-GAL1 or 3 bind to the cell membrane or are internalized by endothelial cells, HRMEC were incubated with biotinylated hr-GAL1 or 3 for 2 h and were visualized by Alexa488 coupled streptavidin. For biotinylated hr-GAL-1 as well as 3 a moderate but specific signal was observed adjacent or colocalized to that of the monocarboxylate transporter-1 (MCT-1), which is a marker for the cell membrane (Fig 1C). Since no fluorescence was detected in the focal plane of the cytoplasm (Fig 1C), our data strongly suggest that both hr-GAL1 as well as 3 bind at the cell membrane but will not be internalized by microvascular endothelial cell.

Further on, we investigated by which mechanisms microvascular endothelial cells could decrease their intracellular galectin-1and -3 levels. To this end, HRMEC were incubated with hr-GAL1 or 3 with or without an additional treatment with epoxomicin, an inhibitor of proteasomal degradation, or brefeldin A, which inhibits exocytosis, for 12 h.

**Fig 2:**
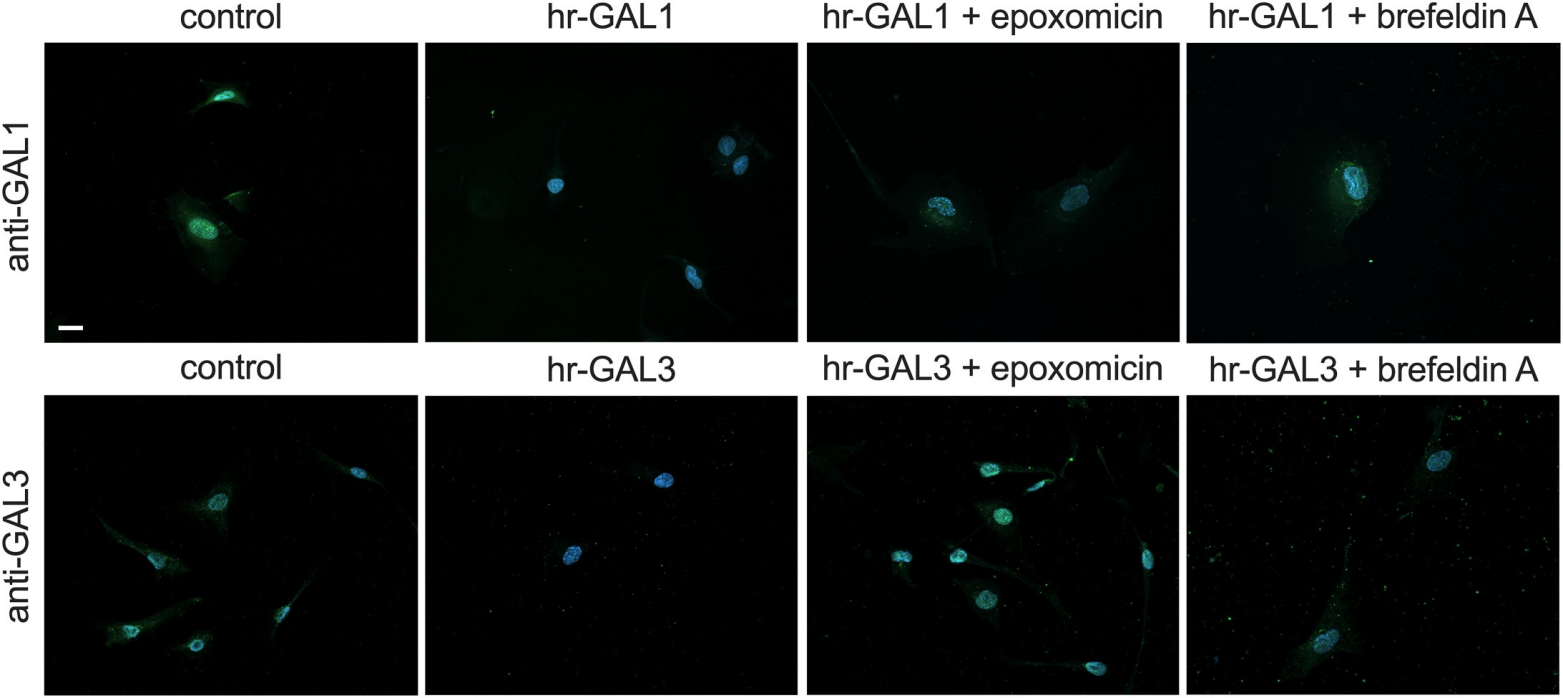
Hr-GAL1 and 3 decreases intracellular galectin-1 and -3 level via exocytosis and proteasomal degradation. Immunostaining of HRMEC for galectin-1 and -3 after incubation with 120 μg/ml hr-GAL1 or 3 with or without 5 μM epoxomicin, an inhibitor of proteasomal degradation, or 10 μM brefeldin A, an inhibitor of exocytosis, for 12 h. Magnification bar, 20 μm; blue, Hoechst staining.

As described before, treatment of HRMEC with hr-GAL1 or 3 markedly decreased the intracellular levels of galectin-1 and -3 (Fig 2). In contrast, following incubation of HRMEC with hr-GAL1 and epoxomicin or brefeldin A an increased signal for galectin-1 was detected when compared to cells with a solitary treatment with hr-GAL1 (Fig 2). Homologous results were obtained after treatment of HRMEC with hr-GAL3 and epoxomicin or brefeldin A (Fig 2). Overall, our data strongly suggest that the reduction of intracellular galectin-1 and -3 after treatment with hr-GAL1 or 3, respectively, could be mediated via both exocytosis and proteasomal protein degradation.

### hr-GAL1 and 3 block cell proliferation of HRMEC *in vitro*

Since proliferation of microvascular endothelial cells is a hallmark of angiogenesis, we investigated if high concentrations of hr-GAL1 or 3 have proliferative or inhibitory effects on HRMEC proliferation *in-vitro*.

**Fig 3:**
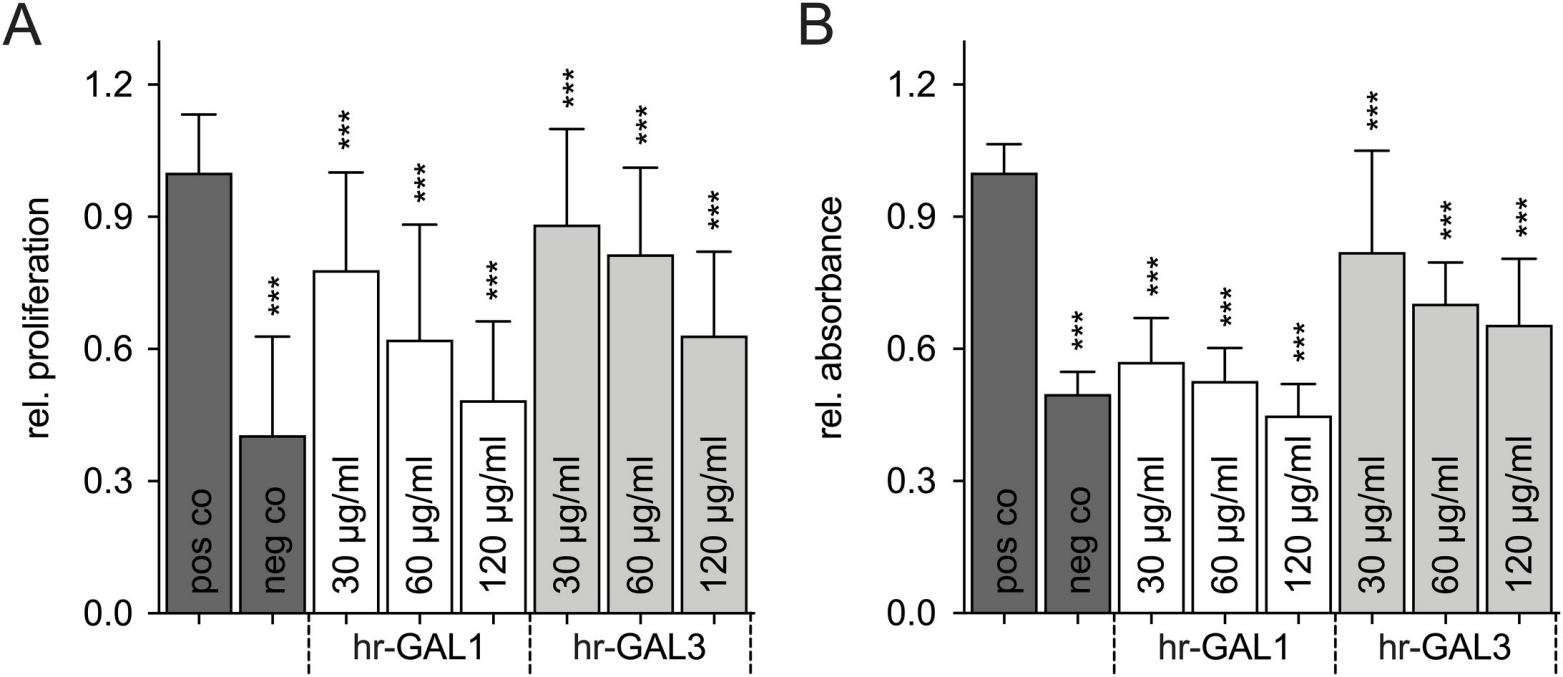
hr-GAL1 and 3 block cell proliferation of HRMEC *in vitro*. BrdU ELISA (A) and WST-1 (B) assay after treatment of HRMEC with 30, 60, and 120 μg/ml hr-GAL1 and 3 for 48 h (BrdU) as well as 72 h (WST-1). *co vs all hr-GAL1 and 3 concentrations, ***p<0.001; BrdU n = 40 of 5 independent experiments; WST-1, n = 32 of 4 independent experiments.

After treatment of HRMEC with human recombinant galectins in growth factor supplemented cell culture medium for 48 h, a significant, dose-dependent decrease of cell proliferation up to 52% as well as 37% after incubation with 120 μg/ml hr-GAL1 or 3, respectively, was detected by BrdU ELISA compared to cells cultured under supplemented conditions (Fig 3A), hereinafter referred to as positive control (pos co). When compared to HRMEC, which were incubated in unsupplemented cell culture medium, hereinafter referred to as negative control (neg co), a 1.3-fold increase for hr-GAL1 (120 μg/ml) and 1.5-fold increase for hr-GAL3 (120 μg/ml) was observed (Fig 3A), whereas the positive control displayed a 2.4-fold increase when compared to negative controls (Fig 3A).

To validate our data of blocked proliferation of HRMEC following incubation with galectins with an independent analysis, the intracellular amount of NADH was measured by a WST-1 assay. After treatment of HRMEC with hr-GAL1 or 3 for 3 days, a dose-dependent decrease in WST-1 turnover was observed. The antiproliferative effect was most prominent at a concentration of 120 μg/ml hr-Gal1 and 3 and led to a highly significant reduction to 45% and 65%, respectively, when compared to the positive control (Fig 3B). Overall, both analyses strongly suggest an anti-proliferative effect of high concentrations of hr-GAL1 and 3 on HRMEC *in vitro*.

### hr-GAL1 and 3 inhibit cell migration of HRMEC *in vitro*

For vascularization of tissues by angiogenesis, microvascular endothelial cells move away from preexisting vessels to form new vasculature. To investigate the migrative potential of HRMEC following incubation with hr-GAL1 or 3, a scratch wound healing assay and time-lapse microscopy were performed.

**Fig 4:**
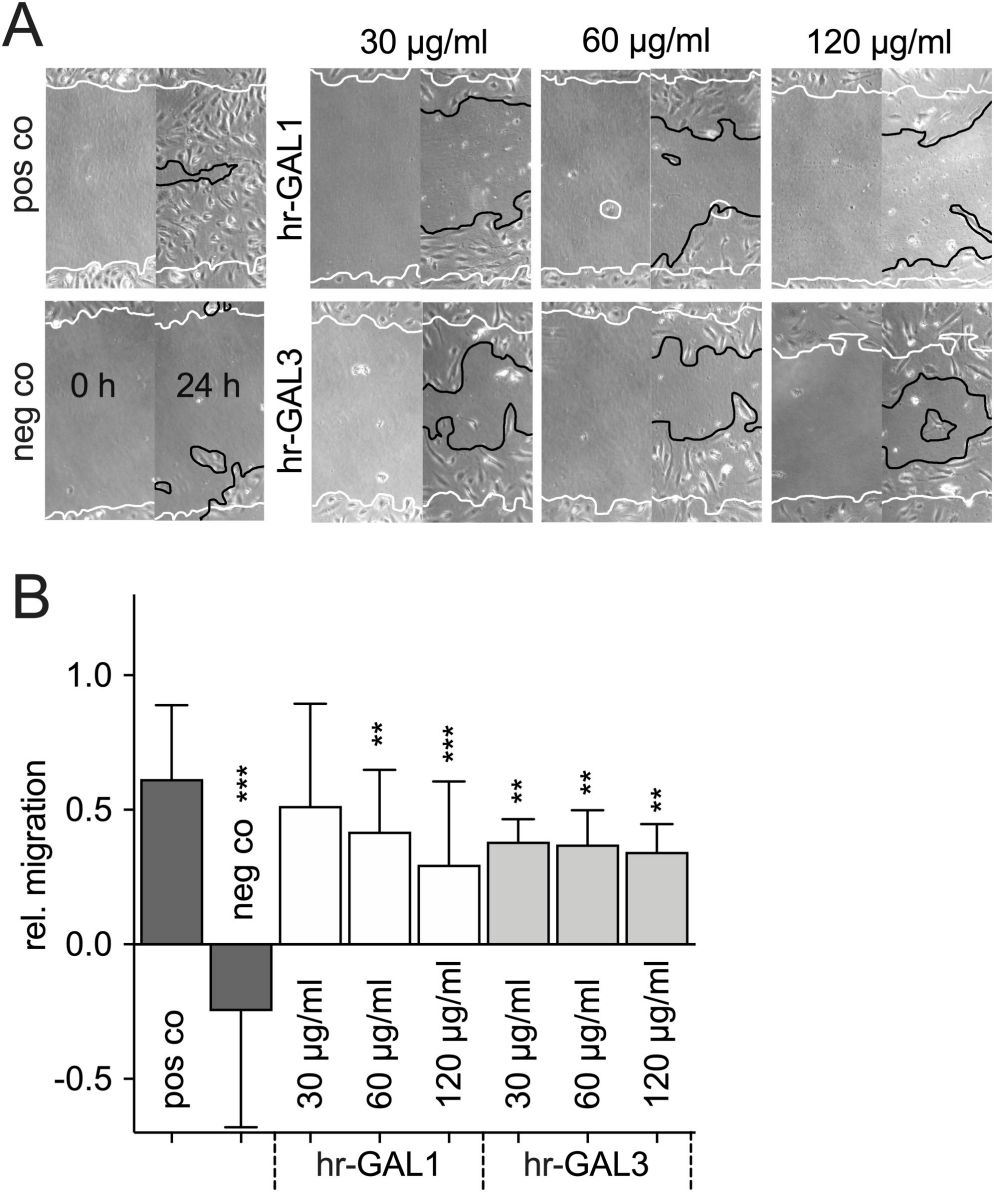
hr-GAL1 and 3 inhibit migration of HRMEC *in vitro*. Scratch migration assay (A) and quantification (B) of HRMEC after incubation with 30, 60 and 120 μg/ml hr-GAL1 or 3 for 24 h. The left half of all images shows the wounded area immediately after scratching (0 h), the right half the same region after incubation for 24 h. *positive control (pos co) vs treated cells or negative control (neg co), ***p<0.001; n = 20 of 4 independent experiments.

Almost the entire wounded area was covered by HRMEC after incubation for 24 h in supplemented cell culture medium, whereas following cultivation of the cells in cell culture medium without supplement, only a few cells migrated into the wounded area (Fig 4A). Following treatment with 120 μg/ml hr-GAL1 or 3 in supplemented cell culture medium, the re-colonized area was significantly lower by approximately 31% and 44% for hr-GAL1 and 3, respectively, when compared to the positive control (Fig 4B).

**Fig 5:**
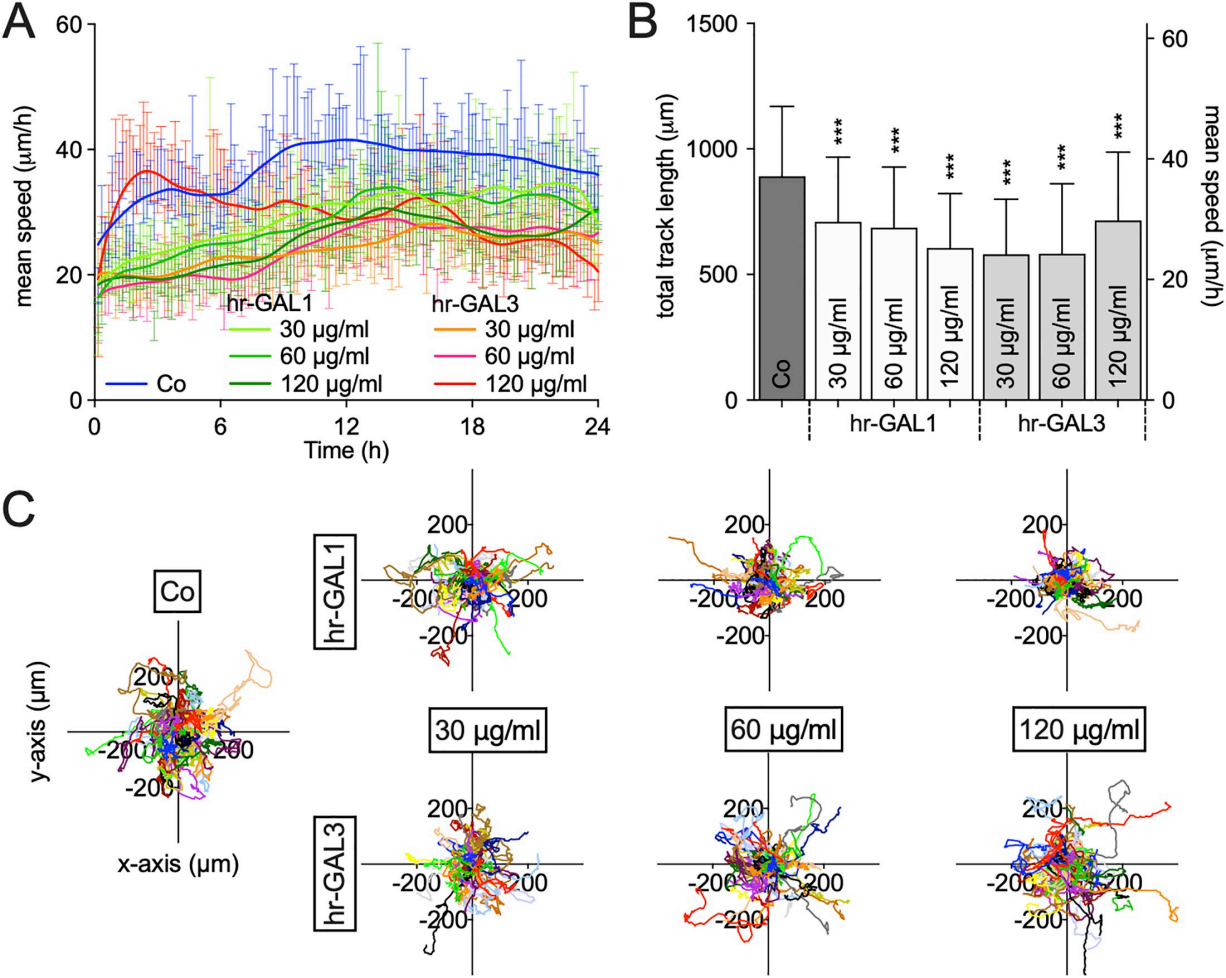
hr-GAL1 and 3 slow down cell velocity of HRMEC *in vitro*. Time lapse microscopy documentation of mean cell migration velocity at the documented time intervals (A), the overall mean velocity after incubation (B) and the single-cell superimposed cell tracks (C) during 24 h of incubation with 30, 60 and 120 μg/ml hr-GAL1 or 3. Each color in (C) represents one superimposed cell track. *co vs all hr-GAL for velocity, ***p<0.001, n = 60 of 3 independent experiments.

Since wound closure in the scratch migration assay not only relies on migration, but also on proliferation and identifies collective cell migration, time-lapse microscopy was performed to track single cell migration properties following incubation with hr-GAL1 or 3. The mean migration velocity was quantified from the manually followed cell tracks. Both galectins showed a significant reduction of the mean cell velocity throughout the time of documentation at each measured time interval (Fig 5A). The overall cell migration velocity after incubation with 120 μg/ml of either hr-GAL1 or 3 for 24 h was reduced by approximately 32% and 21%, respectively, compared to positive control cells (Fig 5B). The detailed analysis of each single cell track showed that hr-GAL1 and 3 hindered the directional persistence of the cells in comparison to the untreated positive control, leading to a decreased and random migration behavior in the superimposed cell tracks (Fig 5C).

### Hr-GAL1 and 3 inhibit adhesion of HRMEC

For migration of microvascular endothelial cells and maturation of new formed vessels, normal adhesion is essential. To analyze the effect of hr-GAL1 and 3 on the attachment of HRMEC cells, a cell adhesion assay was performed.

**Fig 6:**
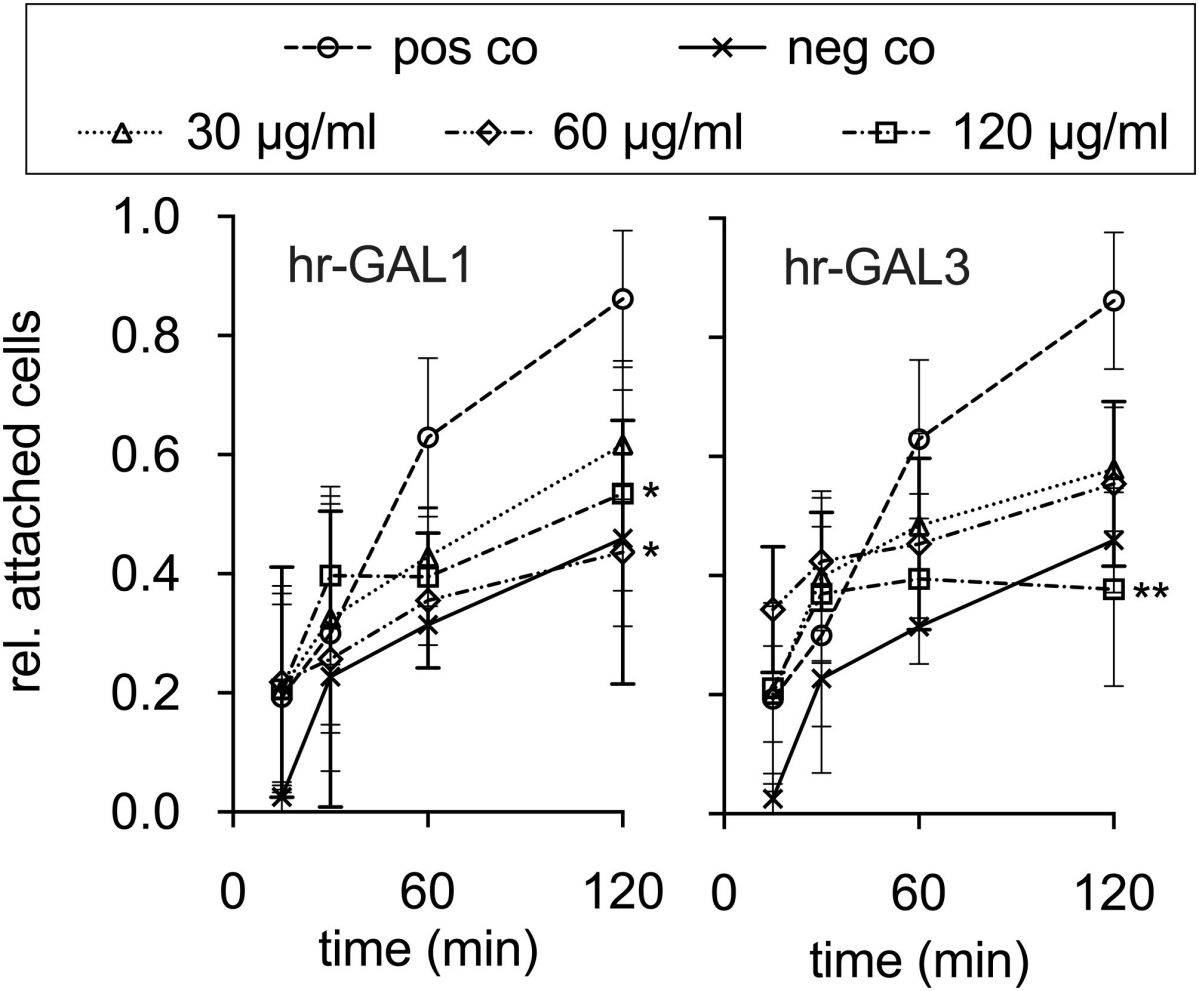
hr-GAL1 and 3 inhibit attachment of HRMEC *in vitro*. Cell adhesion of HRMEC in the presence of 30, 60 and 120 μg/ml of hr-GAL1 and 3. Adherent cells were quantified after 15, 30, 60 and 120 min. *positive control (pos co) vs hr-GAL1 or 3; *p<0.05, (vs hr-GAL1 60 μg/ml: p = 0.021, vs hr-GAL1 120 μg/ml: p = 0.036) **p<0.01(vs hr-GAL3 120 μg/ml: p = 0.01); n = 9 of 3 independent experiments.

After 2 h, more than 80% of HRMEC were attached to the cell culture dish when cultured in supplemented cell culture medium without galectins. However, following incubation of HRMEC with hr-GAL1 or 3 for 2 h, a dose-dependent reduction of cell adhesion of more than 50% was observed for both galectins (Fig 6), strongly suggesting that hr-GAL1 and 3 can block adhesion of HRMEC.

### Hr-GAL1 and 3 inhibit formation of capillary-like structures

Since we could show that high amounts of galectin-1 and -3 can block proliferation, migration and adhesion of HRMEC, we investigated if hr-GAL1 and 3 also inhibit formation of capillary-like structures *in-vitro*.

**Fig 7:**
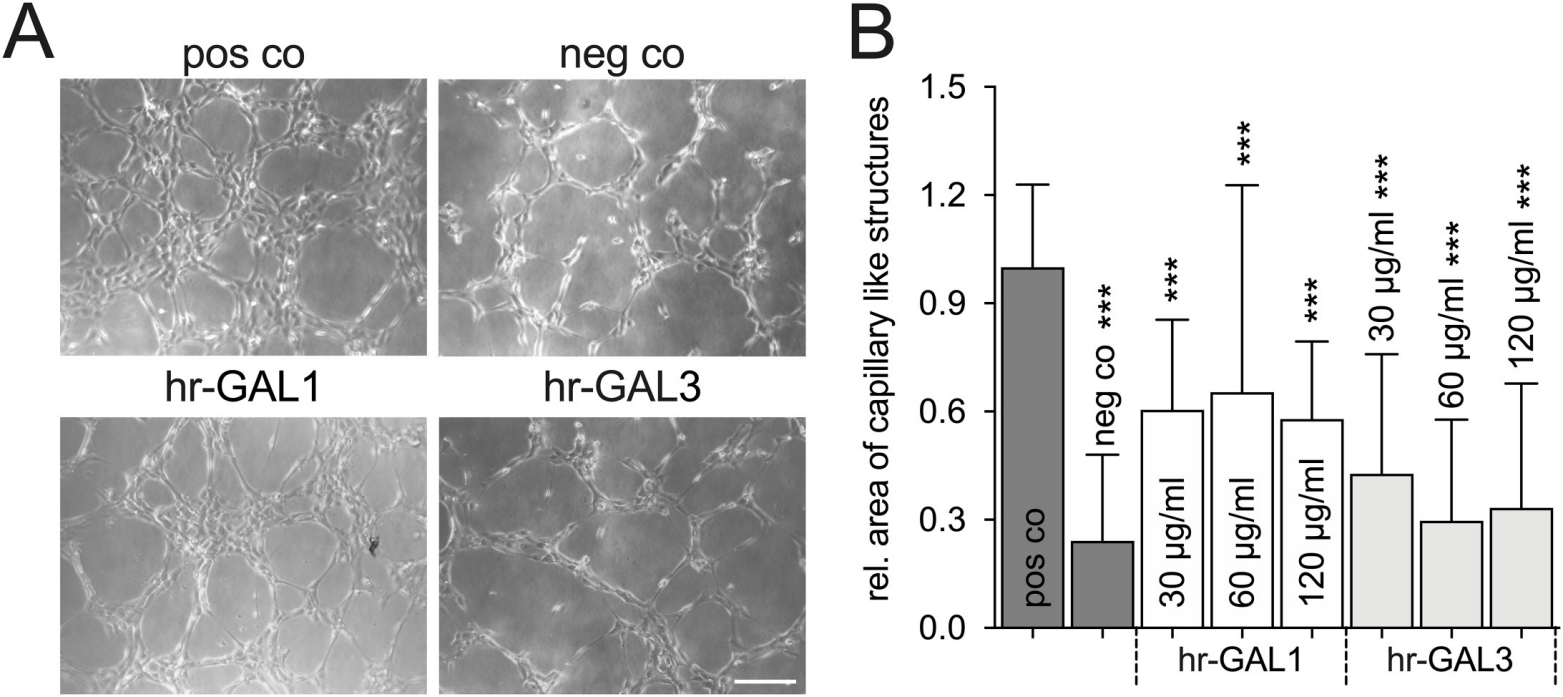
Hr-GAL1 and 3 inhibit formation of capillary-like structures *in vitro*. A. Formation of capillary-like structures after incubation of HRMEC in basal (neg co) or cell supplemented culture medium without (pos co) or with 120 μg/ml rh-GAL1 or 3 for 4h. B. The area, which was covered by capillary-like structures with a width of more than 30 μm, was quantified after incubation with hr-GAL1 or 3 for 4 h and plotted as relative area of capillary-like structures. *positive control (pos co) vs hr-GAL1 and 3; *** p<0.001; n = 24 of 4 independent experiments; magnification bar, 200 μm.

After incubation of HRMEC seeded on an extracellular matrix gel in supplemented cell culture medium for 4 h, a dense network of capillary-like structures with a width of more than 30 μm formed compared to the negative control cells without supplementation of the cell culture medium. Treatment of HRMEC with several concentrations of hr-GAL1 and 3 for 4h substantially reduced the area, which was covered by capillary-like structures. Quantification showed that the area of capillary-like structures was significantly reduced to approximately 60% as well as 30% after treatment with hr-GAL1 and 3, respectively, when compared to the positive control (Fig 7A and 7B).

### Hr-GAL1 and 3 have no toxic effect on HRMEC

Incubation of HRMEC with hr-GAL1 and 3 in high amounts indicated an inhibitory effect on neo-vessel formation *in-vitro*. To rule out that our observations are mediated through any toxic effects of hr-GAL1 or 3, an ELISA against histone-DNA complexes and live/dead staining of HRMEC was performed.

**Fig 8:**
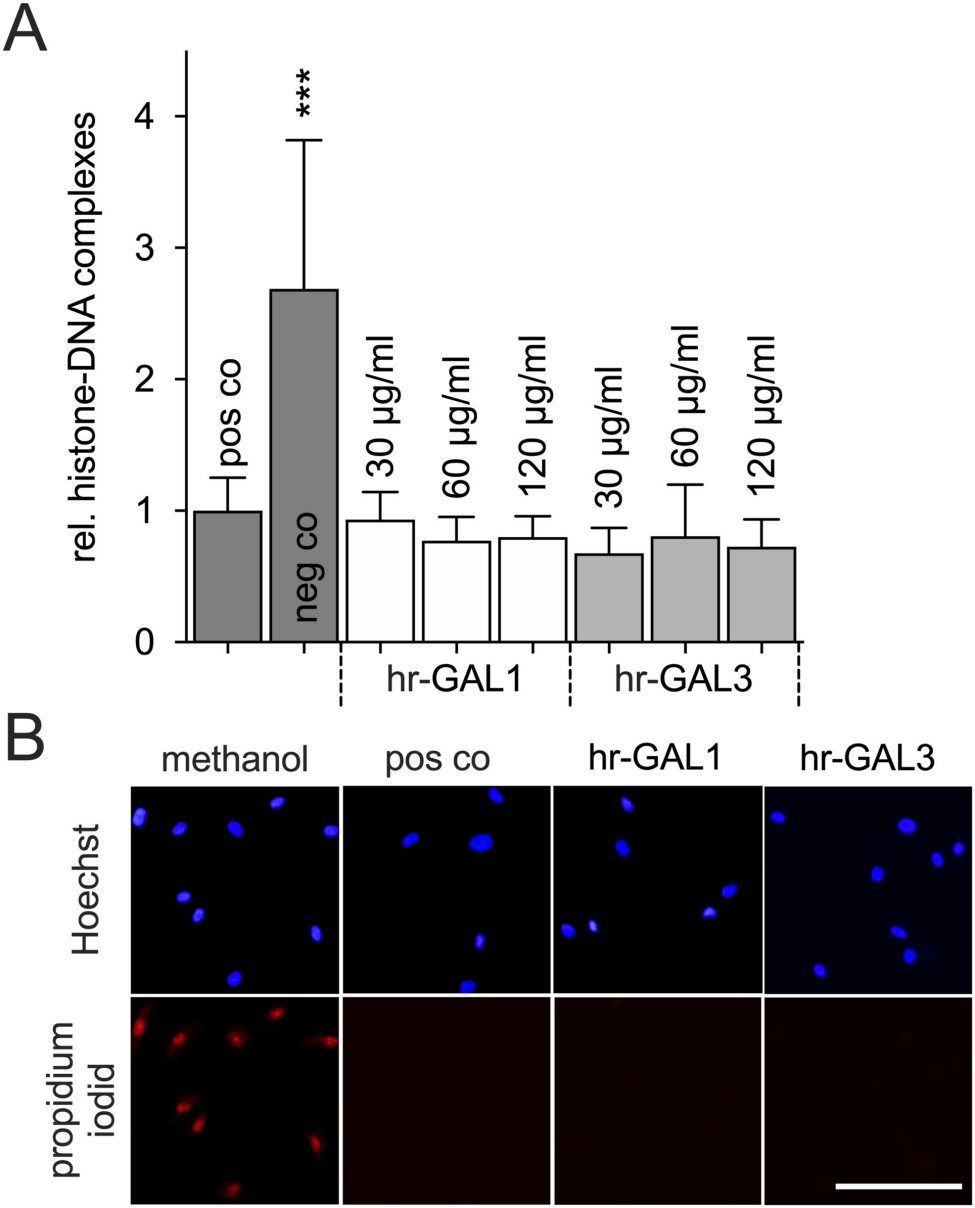
Hr-GAL1 and 3 have no toxic effects on HRMEC. A. Relative level of histone-DNA complexes after incubation of HRMEC in cell culture medium without (neg co) or with supplements (pos co) and hr-GAL1 or 3 for 72 h. B. Live/dead staining of HRMEC with Hoechst 33342 (blue) and propidium iodide (red) after 72 h treatment with 120 μg/ml hr-GAL1 or 3. *pos co vs neg co; ***p<0.001; n = 12 of 3 independent experiments; magnification bar, 75 μm.

Following incubation of HRMEC in cell culture medium without supplements for 72 h, a significant increase of histone-DNA complexes was detected compared to cells which were cultured with supplements. However, following treatment of HRMEC with hr-GAL1 or 3, no changes in the amount of histone-DNA complexes were observed when compared to cells which were incubated in supplemented cell culture medium (Fig 8A).

Using live/dead staining, several propidium iodide positive nuclei were observed following treatment of HRMEC with methanol, which serves as the positive control. However, only a few propidium iodide positive cells were seen in HRMEC cultures after incubation in supplemented cell culture medium with or without hr-GAL1 or 3 for 72 h (Fig 8B). Overall, our data strongly suggest that exogenous galectin-1 or -3 in high amounts mediate no toxic effect on HRMEC.

### Hr-GAL1 and 3 activate Wnt/β-catenin signaling in HRMEC

**Fig 9:**
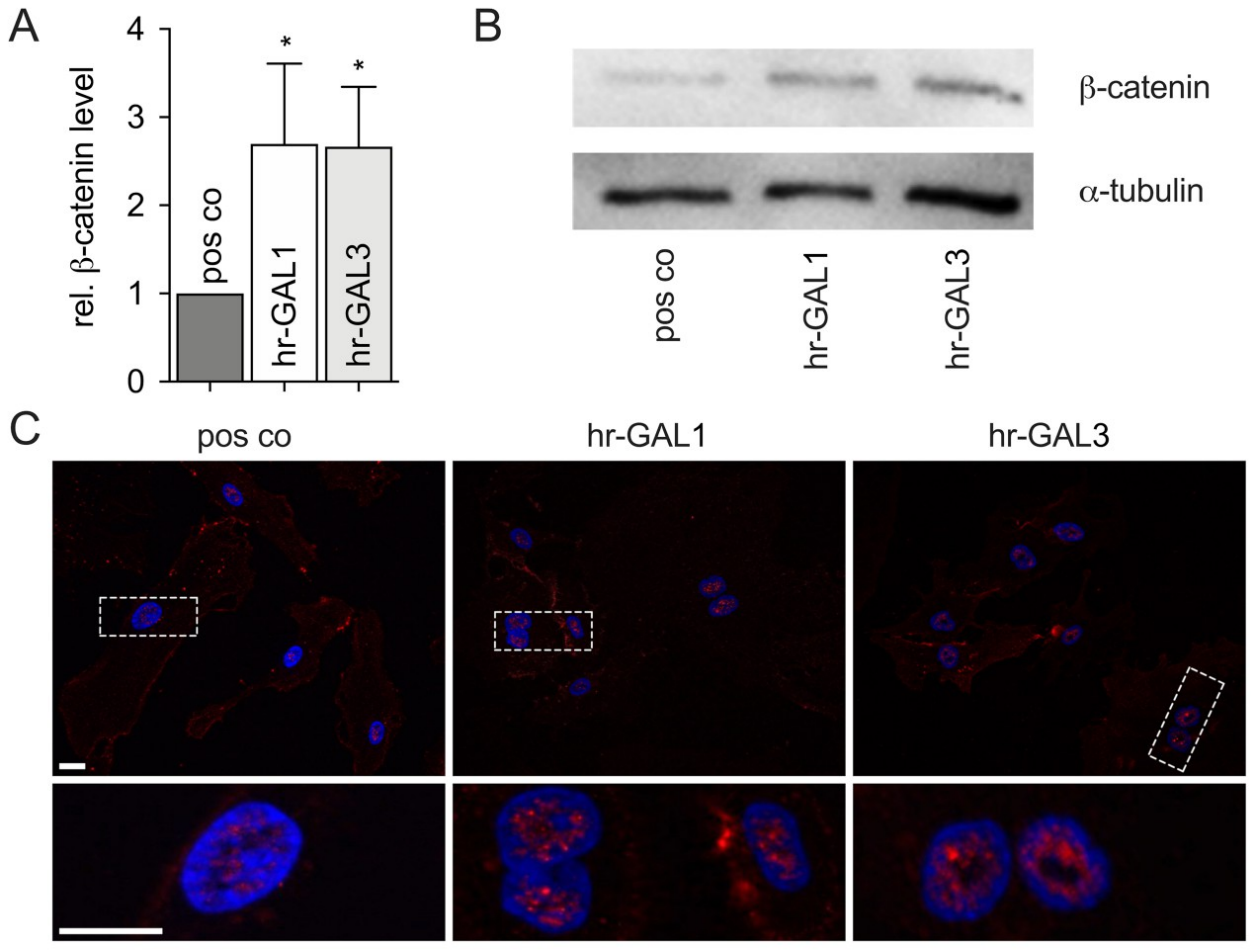
Hr-GAL1 and 3 activate Wnt/β-catenin signaling in HRMEC *in vitro*. A, B. Western blot analysis (B) and densitometry (A) for β-catenin on proteins from HRMEC after incubation with 120 μg/ml hr-GAL1 or 3 for 24 h (*co vs hr-GAL1: p = 0.019, *Co vs hr-GAL3: p = 0.021) *p<0.5; n = 3 of 3 independent experiments. C. Immunofluorescent staining for β-catenin (red) after incubation with 120 μg/ml hr-GAL1 or 3 for 24 h. Magnification bars, 20 μm; blue, Hoechst staining.

Since the retinal canonical Wnt/β-catenin signaling is crucial for its vascularization and galectins can activate this pathway [47–49], we analyzed if hr-GAL1 and 3 could mediate their antiangiogenic effects by modulating the Wnt/β-catenin pathway. In untreated HRMEC, a specific band for β-catenin was detected by western blot analysis. In contrast, after incubation of HRMEC with 120 μg/ml hr-GAL1 or 3 for 24 h, an intense signal of β-catenin was detected (Fig 9B). Densitometry shows a significant increase of β-catenin protein levels of 2.7 ± 0.9 and 2.7 ± 0.7-fold after treatment with hr-GAL1 and 3, respectively, when compared to untreated control cells (Fig 9A).

To further investigate if β-catenin translocates into the nucleus and hereby activates Wnt/β-catenin signaling, immunohistochemical staining of HRMEC was performed.

In untreated cells, a specific staining for β-catenin was predominately observed at the cell membrane and in the perinuclear cytoplasm. Only a weak staining was seen in the nucleus. Following incubation of HRMEC with hr-GAL1 and 3 for 24 h, an enhanced signal for β-catenin was detected in the nucleus of the cells in addition to the perinuclear staining, strongly suggesting that hr-GAL1 and 3 activates canonical Wnt/β-catenin signaling in HRMEC (Fig 9C).

## Discussion

We conclude that exogenous recombinant galectin-1 and -3 have the distinct potential to block angiogenic properties of HRMEC *in vitro* an effect that could be mediated via a depletion of endogenous intracellular galectin-1 and -3. Our conclusions are based upon (1) the observation that both galectin-1 and -3 in high amounts can inhibit proliferation, migration, adhesion and formation of capillary-like structures of human retinal microvascular endothelial cells, (2) the finding that exogenous galectin-1 or -3 mediate no toxic effects on the cells, (3) the capability of exogenous galectin-1 and -3 to activate essential angiogenic signaling pathways such as Wnt/β-catenin signaling and (4) the observation that recombinant galectin-1 and -3 induces a depletion of endogenous intracellular galectins.

In angiogenesis—during development as well as in pathological retinal neovascularization—microvascular endothelial cells of preexisting vessels adopt a tip cell phenotype to migrate into ischemic tissue towards a gradient of angiogenic growth factors. Adjacent microvascular endothelial cells proliferate and trail behind the tip cells to form and elongate the new vessel (for review see [50]). To investigate the pro- or anti-angiogenic potential of substances *in vitro*, several assays have been established for cultured microvascular endothelial cells including proliferation, migration, adhesion or formation of capillary-like structures [51]. The treatment of HRMEC with hr-GAL1 and 3 clearly indicated an inhibitory effect of both galectins on angiogenic properties of HRMEC, suggesting an anti-angiogenic effect. The role endogenous galectine-1 and -3 have on microvascular endothelial cells *in vivo* and *in vitro* is contradictory and depends on multiple factors like microenvironment, endothelial cell source or galectin concentration (for review see [52]). In line with our observations, exogenous galectin in higher amounts showed an inhibitory effect on proliferation of endothelial cells, fibroblasts, HEP 2 carcinoma cells, U2 OS osteosarcoma cells or neuronal progenitor cells in previous studies [35, 53, 54]. A potential explanation for the antiangiogenic effects of hr-GAL1 and 3 on HRMEC could be a toxic effect of the recombinant proteins, which we could rule out by life/dead staining and anti-histone/DNA ELISA.

As shown before, endogenous galectin-1 as well as -3 mediate their proangiogenic effects at least in part via enhancing the signaling intensity of proangiogenic pathways such as Jagged-1/Notch, VEGF, FGF-2 or Wnt/β-catenin signaling [21, 48, 55]. In line, in HMREC hr-GAL1 and 3 enhance proangiogenic Wnt/β-catenin signaling despite their inhibitory effect on the angiogenic properties of HMREC. For galectin-3, both an activating as well as an inhibiting effect on the canonical Wnt/β-catenin signaling pathway has been reported [56]. As potential mechanism for both galectins an activation of the AKT pathway has been discussed, which in turn can inhibit the β-catenin destruction complex and subsequently increases cytosolic as well as nuclear β-catenin, the central mediator molecule of this signaling pathway [57, 58]. Since AKT signaling is activated by receptor tyrosine kinases it is tempting to speculate that galectin-1 and -3 could mediate this effect by interacting with these receptors. However, the inhibitory effects and the activation of Wnt/β-catenin signaling strongly suggests that exogenous hr-GAL1 and 3 activate and not suppress angiogenic signaling in microvascular endothelial cells. However, the attenuation of angiogenic signaling pathways appears to be unlikely to mediate the inhibitory effects of galectin-1 and -3 on the angiogenic properties of HRMEC *in vitro*.

Galectins are not only located extracellularly, but also in several intracellular compartments including the nucleus, modifying various cellular processes such as apoptosis or proliferation [59–62]. Intriguingly, during development of myoblasts and mammary gland epithelial cells, galectin-1 is predominately localized intracellularly, whereas after differentiation, it is mainly localized on the plasma membrane or in the extracellular matrix [63, 64]. Further on, in ductal mammary gland epithelial cells, the extracellular binding of galectin-1 can draw intracellular galectin-1 out of the cells [64]. In line with these observations, following incubation of HRMEC with hr-GAL1 or 3, we observed that the intracellular level of endogenous intracellular galectin-1 or -3 were markedly reduced. The effect that the extracellular binding of galectins to glycoproteins could lower their intracellular level and hereby their intracellular function was postulated by Johannes and colleagues as a potential regulatory feedback loop [65]. Since endogenous signaling of galectin-1 and -3 promotes mitosis of various cells, it is tempting to speculate if the loss of intracellular galectins could mediate the anti-angiogenic effect of hr-GAL1 and 3 in HRMEC. Several reports of a proangiogenic function of galectin-1 and -3 are based on observations on galectin deficient mice or knockdown in microvascular endothelial cells [55, 66]. Therefore, it has to be considered that not only the loss of extracellular galectin-1 or -3, but also their intracellular lack might contribute to the observed antiangiogenic effects in these galectin loss of function models.

The interaction of cells with the extracellular matrix is one of the key functions for development of capillary-like structures, adhesion and migration, which involves adhesion receptors such as integrins [55]. Depending on the environmental conditions, opposed functions for galectin-1 and -3 on cell migration have been reported for several cell types. The opposed effects are mediated via direct interaction between galectins and the extracellular matrix as well as via cellular receptors [67, 68]. Therefore, it is most likely that hr-GAL1 and 3 in high amounts mediate their inhibitory effects on the development of capillary-like structure, adhesion and migration via interactions with extracellular receptors and the extracellular matrix.

Formation of retinal or choroidal neovascularizations is a severe vision-threatening complication of several diseases such as age-related macular degeneration, diabetic retinopathy or retinopathy of prematurity. In mouse models to investigate retinal or choroidal neovascularizations, the loss of endogenous galectin-1 or -3 leads to a reduced neovessel formation or to a maintenance of the blood-retinal barrier [25, 27, 28, 69]. In contrast, we observed an antiangiogenic effect of exogenous hr-GAL1 and 3 on HRMEC *in vitro*. Opposing effects of galectins depending on several circumstances have been reported previously. For instance, in a mouse model for experimental autoimmune orchitis, the lack of galectin-1 in *Lgals1* deficient mice but also the exogenous application of recombinant galectin-1 caused a reduction in the incidence and severity of the disease [34]. Again, these opposed effects of galectins could be caused by the lack of endogenous intracellular galectins and the potential function of extracellular recombinant proteins leading to homologous effects.

Overall, we conclude that exogenous hr-GAL1 and 3 in high amounts have a distinct potential to block the angiogenic properties of HRMEC *in vitro* and therefore could be a promising approach to prevent retinal neovascularization in several diseases.

## Supporting information

S1 Raw images (PDF)
