# Supplementary material for: Galectin-1 and -3 in high amounts inhibit angiogenic properties of human retinal microvascular endothelial cells in vitro
Source: PLoS One. 2022 Mar 23;17(3):e0265805. doi: 10.1371/journal.pone.0265805 (PMC8942239; doi:10.1371/journal.pone.0265805)
Supplement: S1 Raw images — (PDF) [file pone.0265805.s001.pdf]

Original Western Blot for β-Catenin

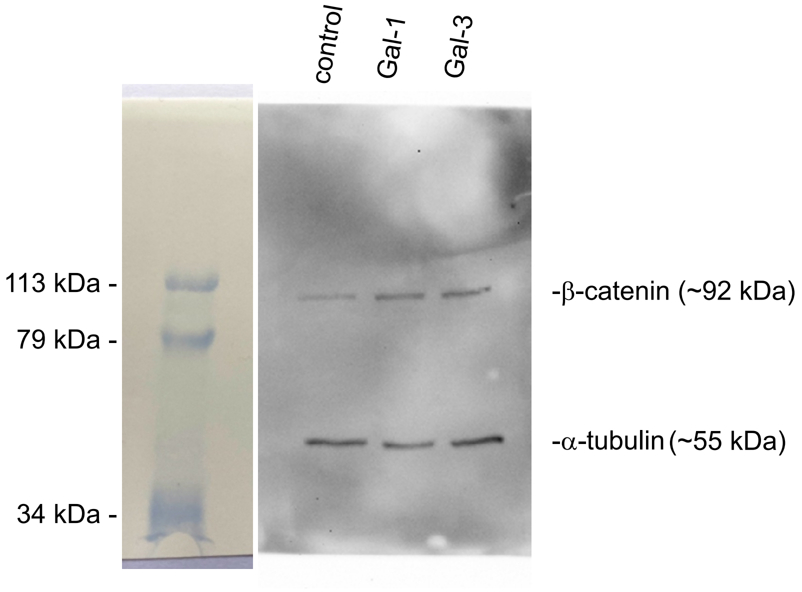

Original western blot after hybridization against β-catenin and α-tubulin.
